# Dialogical meanderings - evaluation framework and learnings from stakeholder involvement in the co-design of a participatory systematic review

**DOI:** 10.1186/s40900-025-00809-w

**Published:** 2025-11-26

**Authors:** G. Ruiz-Perez, U. M. Kraemer, J. Lippert, N. Ullrich, S. von Peter

**Affiliations:** 1 Brandenburg Medical School, Immanuel Klinik Rüdersdorf, Centre of Mental Health – Psychiatry and Psychotherapy, Fehrbelliner Strasse 68, 16816 Neuruppin, Germany; 2 Kellerkinder e.V. Mental Health Self-Advocacy Organization, 12049 Berlin, Germany

**Keywords:** Mental health, Systematic review, Early phase, Participatory research, Mixed method, Collaborative

## Abstract

**Background:**

Internationally, systematic and other review studies are being increasingly conducted in participatory ways. However, there is a lack of knowledge regarding the involvement of various stakeholders in their early pre-protocol stages.

**Aims:**

Given this shortage, this manuscript the participatory preparaton of a systematic participatory, mixed methods review on the reduction and discontinuation of antipsychotic medication.

**Methods:**

The evaluation used both standardized assessments and a focus group to assess 1) the characteristics, knowledge backgrounds, and expertise of the different stakeholders involved, 2) the topics of discussions that were exchanged during the different participatory formats as well as 3) stakeholders’ perceptions.

**Discussion and Practical Implications:**

The various participatory activities involved both users/survivors and researchers with lived experience and/or academic expertise in the field of reduction and discontinuation of antipsychotic medication. They have laid the foundation for the development of the subsequent review design. The participatory exchange was implemented in rather non-directive ways, so that, next to these methodological decisions, a range of other topics could be discussed that iteratively contributed to shaping them. Overall, satisfaction with participation was high. It was concluded that the early phases of a review study are highly suitable for participatory engagement, laying the fundamental grounds for further research. Sufficient time should be provided to substantially anchor the future review design in the stakeholders’ specific and diverse perspectives and positions.

**Patient and Public Contribution:**

The research team was collaboratively staffed by a portion of researchers who were also identified as users or survivors of psychiatric services. The different participatory formats involved a significant number and wide range of diverse stakeholders relevant to the research and practical field of the reduction and discontinuation of antipsychotic medication.

**Supplementary Information:**

The online version contains supplementary material available at 10.1186/s40900-025-00809-w.

## Abstract (key message)

Systematic reviews summarize the studies from a field of research. In many countries, the results of these reviews are decisive for the approval and funding of treatment options. For these reasons, reviews should be undertaken together with the people that are at the center of concern of the object of study to allow them to decide on the questions that will be investigated and the study design used for this purpose.

This manuscript describes the processes and results of the participatory planning of a review on the discontinuation and reduction of psychiatric drugs that are used to treat people with psychotic experiences. Even though these drugs are effective in acute illness phases, they may have harmful long-term outcomes, making the discontinuing and reducing of these medications a pivotal research topic.

**Supplementary Information:**

The online version contains supplementary material available at 10.1186/s40900-025-00809-w.

## Background

During the last few decades, intentions for involvement and participation of (ex-)users of psychiatric services or related so-called stakeholders have increased in the field of mental health research, in some countries growing into a real trend [1–4]. Referring only to two countries that are also relevant in the context of the present study, this trend has been fueled in the United Kingdom since 1996 by various funding bodies or research guidelines, leading to a peak of about 800 peer and user researchers in the second decade of the 21^st^ century working in various national projects [5, 6]. In Germany, developments started much later, the first network being constituted in 2007 [7], followed by the publication of policy papers or strategic frameworks by various scientific communities, public, or funding agencies [8–10], which was complemented by an increasing variety of empirical research based on different research approaches, whereby it is not always clear which participatory practices (participation, involvement, engagement, co-production, etc.) are behind this approach.

What is often forgotten is the emancipatory, often non-institutionalized, research activities that are controlled by single or groups of (ex-)user/survivor researchers, which have produced a wealth of highly insightful knowledge internationally [11–13]. Usually starting from their experiences of discrimination or rights violation in mental health care settings [14, 15], these initiatives represent the (ex-)users/survivors’ strong desire to produce a knowledge base “on our own” [15, 16]. Instead of imposing a unified counter-epistemology, critical debates on mental health (care) have pushed towards various disciplinary sectors, thereby drawing on a variety of perspectives and standpoints [17, 18]. Having already originated in some countries in the late 1980ies, these research strands still exist, even increasing in some cases [13, 17], which reflects both the persistence of the harmful situations criticized and the fact that many of these researchers resist participating in usual mental health research and are not deemed to allow for sufficient agency or to lead to any change or transformation in their lives [11, 17].

This resistance is important to the background of this manuscript, reporting on various participatory activities with (ex-)users/survivors of psychiatric services and other critical experts to conceptualize and prepare a systematic review on the reduction and discontinuation of neuroleptic drugs – the conceptual phase of the PARTANE study [19]. It follows the research questions of how various stakeholders may be involved in the early stages of a review study and how can such processes be evaluated? These questions are pursued as, even though the contribution of involvement to the quality, relevance, and impact of systematic reviews is increasingly recognized [20, 21], there is a lack of knowledge on how to meaningfully do so. This may be due to the overall shortage of review projects that involve, to a larger extent [22, 23], most of which report on these activities in a rather low quality, and some of them being study protocols [24, 25], which makes it difficult to obtain a more detailed understanding of the exact procedures. Furthermore, most papers identified in the field of (mental) health care report on the involvement processes during the whole review process [21–23, 25–29], whereas none concentrate only on the early pre-protocol phases.

Moreover, most review studies do not include any evaluation of their involvement processes, making it difficult to understand how these processes have been perceived by the participating stakeholders or how they have been implemented in detail. Furthermore, most literature on the question of how to evaluate processes of involvement or participation in research is of more general value, instead of providing more precise guidance on how to evaluate in the specific context of a review study [20, 21, 24]. Of the few review studies that have incorporated assessment tools, most have used mixed methods approaches by combining standardized with qualitative assessments, pointing at the complexity of these kinds of evaluations. Thereby, they base their evaluations on different criteria or indicators, indicating the need to adapt these instruments to the specific research context and prove the desiderate for more nuanced examples. Furthermore, most of the related manuscripts are protocols, complicating a clear understanding of the implementation of these evaluative measures and thereby neither, as mentioned above, specifically relates to an early review stage.

Given this background, a full work package of the PARTANE conceptual phase was dedicated to the description and evaluation of the various participatory activities undertaken during this preparatory phase, being at the center of this manuscript, the following two aims: first, the participatory activities of this conceptual phase are described, analyzed, and critically discussed; how they were implemented by the research team and, conversely, made use of and experienced by their participants, to ultimately contribute to more knowledge on how to meaningfully participate in a pre-protocol stage of a systematic review process. Second, the evaluative framework of the PARTANE conceptual phase is presented; by describing and critically discussing the instruments employed and the proceedings undertaken; to enhance, on a more general level, the understanding of how participatory activities may be evaluated in such an early review phase. Whereas the processes of participatory activities are described hereinafter, the outcomes of these processes are presented in a second manuscript currently under development.

## Material and methods

### The context of the study

#### The funding line

The project has been funded by the German Ministry of Education and Research, since 2022 supporting the co-design of clinical or review studies [30] by funding a so-called conceptual phase, in which “users and stakeholders have an active role in the planning and design of a clinical trial or systematic review”. The objective of such a pre-study is “to identify relevant (groups of) stakeholders and ways of reaching them, to find appropriate methods and tools for participation, to develop a role definition, and to collaborate on the design of the subsequent research project” (ibid). This phase terminates with the development of a second funding application for subsequent clinical or review studies, including a concept on how to involve the so-called target group during the implementation phase. Therefore, it does justice to the revised version of the Medical Research Council guidelines on how to assess clinical interventions, placing stakeholder involvement at the center of its evaluative cycle [31], as well as to the guiding framework to review designs, advising to incorporate involvement as early as possible during this process.

#### The PARTANE conceptual study

The idea of a project proposal for the reduction and discontinuation (R&D) of neuroleptic medication (NL) that prioritizes the involvement of collective user/survivor knowledge was introduced by a user researcher who is a co-author (UMK). The proposal was collaboratively developed and written by members of the Co-Lab Mental Health at Medical School Brandenburg (UMK, GRP, SvP), a research group of researchers with and without experience as users of psychiatric services or survivors of psychiatric violence [32]. It was motivated by a recent WHO document that proposes the informed, self-determined use of psychopharmacological medication to be highly relevant to human rights to make decisions concerning one’s own health [33]. Thus, the PARTANE study employs a human-rights oriented theoretical lens that well fits with the diverse approaches and frameworks produced in the field of the (ex-)users’ and survivors’ research and knowledge production mentioned below.

Second, the outcomes of NL are increasingly debated to be questionable, especially their long-term effects [34] and their high burden of treatment [35]. Both the intake of low doses and dose-reduction strategies are advised in various treatment guidelines [36, 37] to minimize the risk of so-called super-sensitivity psychoses following high-dose or long-term intake of NL [38]. Third, knowledge on how to successfully reduce or discontinue these substances is scarce, also due to the usual resistance of psychiatric teams that for decades, following a rather biological rationale, offer mainly psychopharmacological remedies as often the only intervention in the context of psychiatric care [39]. This is the reason why the users/survivors of these services had to find ways to reduce or discontinue NL, usually not being supported in their intentions or attempts to do so [40, 41], causing them to develop individual as well as collective knowledge and mutual support activities within their communities starting around the 1970ies and meanwhile shared via forums, experiential reports, podcasts, or withdrawal guides worldwide. This knowledge is vital source of insight that is, fourth and most importantly to this project, barely being acknowledged in the context of professional discourses – a situation that most recently has been analyzed using a framework of epistemic injustice, representing the main rationale for the involvement of the divers (ex-)users/survivors’ perspectives and positions in the context of the PARTANE study [42].

Given this epistemic background, the overarching goal of the overall PARTANE study is to advance the knowledge base on how to successfully reduce or discontinue NL by placing diverse knowledge, expertise, perspectives, and experiences of (groups of) (ex-) users/survivors (researchers) of psychiatric services at the center of a systematic, multi-step review process. The first step of this process, to which this manuscript refers to – the PARTANE conceptual phase – which lasted from February 2023 until March 2024, aimed at preparing the subsequent steps of the full participatory, systematic, mixed methods review, not yet accomplished, but for which the funding application has just been handed in. In the following review phase, we plan to systematically synthesize the various materials and voices of (ex-)users/survivors’ collective knowledge with evidence from qualitative, quantitative, and mixed methods studies to ultimately identify effective strategies for the successful R&D of NL. To prepare and co-design this endeavor, the preceding conceptual phase aimed to 1) implement a literature search on how to involve diverse stakeholders during this subsequent review process, 2) undertake diverse participatory activities (see 2.3.3) to co-develop the different steps and proceedings of this review process, and 3) evaluate participatory involvement during this early conceptual phase.

No ethical approval was necessary for this conceptual phase and was granted by a waiver of the Ethical Committee of the Medical School Brandenburg. The acronym PARTANE derives from a German title (PARTANE – **Par**tizipatives Co-Design einer systematischen Übersichtsarbeit zur Reduktion und zum Absetzen (**ta**pering) von **Ne**uroleptika bei Menschen mit Schizophrenie-assoziierten Störungen = participatory co-design of a systematic review on the reduction and discontinuation (tapering) of neuroleptics in people with schizophrenia-associated disorders).

### The participatory approach of the conceptual phase

As with the application, the planning and implementation of the PARTANE conceptual phase was carried out with the help of multi-perspectival expertise within the MHB collaborative research team. This team was staffed by researchers who integrated scientific knowledge with clinical knowledge or lived experience and epistemic standpoints. More specifically, the team was staffed by two (male) psychiatrists (SvP and GRP) and two (female) user/survivor-researchers (UMK and JL), one of the latter taking on the role of the participation coordinator (JL), organizing all participatory activities described below. Thus, at the level of the research team, individual and collective experiences of having reduced/discontinued NL, or having supported these processes, were continuously present, at times leading to a bifurcation of positions and understandings within the research team. Further, all four researchers had completed a degree in either social anthropology, philosophy, or sociology, making the collaboration also to an inter-disciplinary one, and shaping our shared understandings of the material. Further, one of the psychiatrists had also been contributing to a Berlin-based Trialogue initiative that, since 2019, has been critically exploring the impact and harms of psychopharmacological medication from the various experiential perspectives of (ex-)users, of caregivers as well as of mental health care practitioners. The two researchers of our research team with own experiences with the R&D of NL, further, had coordinated a mental health advocacy project (“Landschaftstrialog”) on how to practically apply human rights in mental health and social services in Germany, shaping both the analytic perspectives and ways of implementing the different participatory formats also in the current project.

Based on this knowledge and insights and considering the different interests and expertise of the participants, two major strands of involvement were implemented in the PARTANE study, each bringing together individuals from Germany, the Netherlands, the UK, and, to a smaller extent, the US. ***Strand 1*** aimed at the meaningful participation of (ex-)users/survivors of psychiatric services, contributing to both individual and collective knowledge of the R&D of NL, as well as supporting others in their attempts to reduce or discontinue. These participants were involved via three participatory workshops ( = WS), offline one-day/eight-hours each, in Berlin (in German language in August 2023), Utrecht, and London (in English language in September 2023), with an average of 12 participants each. The first strand also included a two-hour expert interview ( = EI) in August 2023 that were not able to attend the German WS and, further, two participatory discussion groups ( = DG) that involved eight (offline) and 33 (online) self-help representatives of the German Network ‘’Critical Psychopharmacological Trialogue’’ (September/December 2023) that have exchanged various perspectives around the R&D of NL and other psychopharmaceutical drugs during the last four years by now [43]

***Strand 2*** involved the contributions of a multi-expert advisory board ( = AB), which consulted on the developments and decisions taken throughout the conceptual phase, also contributing other forms of knowledge. Between six and nine participants – researchers with and without their own experiences of the R&D of NL, as well as developers of self-help programs or guides for supporting these processes, or individuals integrating both kinds of knowledge, as shown in more detail in the results section – joined four two-hour online meetings in April, May, October, and November 2023. Meetings 1 and 2 at the beginning of the year 2023 concentrated on the challenges of a participatory review. In meetings 3 and 4, central findings from WS and DG were discussed, as well as their implications for the review study. All members were invited to meetings, whereas the participation of most members was not continuous for reasons of time.

### Reaching out and sampling and selection of participants

As mentioned above, in all three WS, (ex-)users/survivors of psychiatric services and experts by experiences took part, who have gained individual and collective knowledge of taking as well as reducing/discontinuing NL over years. For conceptual reasons and reasons of feasibility, the selection process in all three countries differed: in Germany, we used our personal and collective knowledge of mental health users and survivors that have been active in advocacy and self-help surrounding the R&D of NL. We also approached the national self-help and advocacy organization of users and survivors in Germany (BPE e. V.), another Berlin-based advocacy organisation on the rights of people with mental health impairments (Kellerkinder e.V.), the already mentioned initiative ‘’Critical Psychopharmacological Trialogue’’, the initiative “Psychexit” who had organized seven expert-rounds on diverse subjects in relation to psychotropic drugs, and a German informal online network of (ex-)users/survivors who support each other during their withdrawal processes to suggest potential participants with individual and collective experience of R&D of NL. If potential candidates matched our profile, they were contacted personally. We restricted our search to a maximum of 10–12 participants to allow for meaningful dialogue on potentially sensitive experiences. Interestingly we learned that not all people who fit our profile were interested in participating. Some felt the subject could trigger too painful memories, few felt uncomfortable to talk about their experiences in an ad-hoc-group and lose their anonymity in the online network. Finally, 12 experts-by-experience from all age groups agreed to participate with some travelling several hundred miles to do so. As mentioned above, a two-hours expert interview was organized with two BPE experts that were not able to attend at the WS but have supported people during the R&D of psychotropic drugs for many years.

Differently from Germany, we had no personal contacts to the different (ex-)user/survivor groups and networks in the Netherlands. Various approaches to engage user/survivor organizations for the organization of the WS failed, also due a shortage of time. In the end, the selection of participants and organizational hosting of the WS in the Netherlands was taken over by a senior mental health researcher, who has been supportive in promoting user/survivor research and surrounding networks since years. This experience allowed him to recruit an interesting group of a total of ten (ex-)user/survivors and service user researchers with own experience of withdrawal of NL from various parts of the country. The same was true for the WS in the United Kingdom: again, we had no personal contacts to British user/survivor groups. We thus approached the independent Survivor Researcher Network ( = SRN) for a partnership to sample experts-by-experience. Our team was hugely impressed by the SRN board members’ commitment during several online meetings to discuss from which regions to recruit as well as questions of accessibility and confidentiality. The SRN board members persisted to reach the widest possible diversity of participants, investing extra efforts to contact people from racialized minority groups, some of which declined as they did not want to participate as a tokenist individual due to increase the inequality in numbers of participants. Thus, even the most committed efforts can’t change, for a workshop, the power imbalances present in society and thus in involvement. We wish to at least make this societal injustice transparent in our reporting.

The participants of the AB were chosen one by one by the collaborative research team based on their critical expertise in the field of R&D of NL, their research impact, and/or their reputation among the (ex-)user/survivor movements or researchers.

### The evaluative framework of the conceptual phase

The evaluation of the conceptual phase only focused on the AB and WS meetings, as they, in comparison to the EI and DG meetings, took place recurrently, thus making an aggregated analysis of data possible – and, in comparison to the DG meetings, for research purposes only, thus allowing for formal assessments alongside ethical standards.

Based on the literature indicated in the introduction and further sources focusing on the evaluation of involvement in the context of clinical studies or other research designs [43–47], the evaluative framework was developed by two of the authors (GRP, SvP). As advised, a mixed methods design was chosen to render justice to the complexity of the research phenomenon under study. Evaluative items or questions were either taken out or derived from the context of other studies [43–47] or they were adapted or added in relation to the design specificities of our conceptual study. Following existing frameworks, one part of the assessment focused on the characteristics, knowledge backgrounds, and expertise of different participants from the two strands mentioned above (the “who” – participants). A second part aimed at providing for an overview of the topics of discussions that were exchanged to allow, in a second step, for a comparison between the different participatory formats used (the “what” – contents). The third part of the evaluation analyzed how the stakeholders have experienced and perceived the processes of involvement (the “how” – perceptions). In all three steps, pragmatic reasoning and the limited resources of the only one-year PARTANE project phase constrained the quantity of research tools and items employed, also leading to the decision not to evaluate the impact (“for which purpose”) of involvement, the project lasting for one year only, and not being sufficiently staffed for such an endeavor.

#### Evaluation of participants

To understand more about the type of expertise that contributed to the AB and WS formats, as well as the knowledge backgrounds and sociodemographic characteristics of their participants, an online survey link was sent to them with open and standardized questions to be filled in. As shown below (Table 1), next to the specification of gender and age, the participants were asked to specify their professional and experiential perspectives in reference to their years of experience with processes of the R&D of NL. These items were sent to AB and WS participants only, as the everyday nature of the participatory DG, not being undertaken solely for research purposes, made such an evaluation unfeasible.

**Table 1:** The various backgrounds of both WS and AB participants returned by 20 out of a total of 34 ws participants and all ab participants. Multiple answers were possible

		WS (N = 20)	AB (N = 12)
**Perspectives**			
	User/Survivor	19	3
	Patient Representative	3	3
	Family Member/Informal Caregiver	5	3
	Researcher	9	12
	Political Work/Advocacy	9	5
	Peer Support	8	3
	Staff Member	3	5
	Other	2	3
**Years of experience with this topic**			
	< 6 months	1	0
	1–2 years	1	0
	3–4 years	2	2
	> 5 years	16	10
**Gender**			
	Female	11	3
	Male	8	9
	Diverse	1	0
**Mean Age**			
		51,25	57,4
**Experience of Withdrawal**			
	No	2	X
	Yes	18	X
	Abrupt discontinuation	7	X
	Other ways to withdraw	11	X

#### Evaluation of contents

The discussions of the WS and AB meetings were audio-recorded, transcribed, and content analyzed [48] with the aim of summarizing the topics discussed in each of these formats to enable a quantitative comparison between them in the second step. For this purpose, a coding system was developed by three members of the research team (GRP, JL, and SvP) based on intensive discussions of the transcript material and allowing for a subsequent deductive coding process of all transcripts by two team members (GRP and NU). Subsequently, overarching themes were developed to cluster the groups of codes and categories. The number of codes per category and cluster was counted, and the differences between participatory formats were descriptively analyzed.

#### Evaluation of perceptions

The evaluation of the participants’ perceptions was carried out using both open and standardized questions. After each AB/WS meeting, an online link was sent to the participants to evaluate their perception, for example, did the participants have enough time to prepare for the meetings; was the purpose of the meeting clear enough to them; was the communication of the research team perceived to be clear and transparent; were the participants’ voices involved deemed to be sufficiently diverse and/or from marginalized/vulnerable backgrounds; were the participants able to express their views freely; did they experience that their perspectives were taken seriously by other participants and/or the research team; and were they able to make a valuable contribution, etc.? The full questionnaire can be found in the electronic annex (e3). The quantitative data was analyzed descriptively, the qualitative data was merged with the data from the focus groups below and analyzed using a content analysis method [49]. This survey was not carried out with the participants of the participatory DG because of the shortage of these meetings, making their systematic evaluation pointless.

A focus group was implemented during the last month of the project to understand the participants’ perceptions in more detail and nuances. As the EI, DG, and WS formats involved participants only once, this part of the evaluation concentrated on members of the AB that had participated in at least two or more AB meetings. In addition, three of these individuals participated in each participatory WS so that they could compare their perceptions of participating in both the WS and AB participatory formats. The focus group material was decoded inductively using a content analysis method [50] and the software MAXQDA by one member of the research group (GRP), yet also deductively using the categories previously produced to decode the contents of the various participatory formats.

## Results

As stated above, the evaluative results of the participatory processes of our pre-review PARTANE conceptual phase study are at the center of this manuscript, whereas the outcomes of the conceptual phase will be presented in a second manuscript currently under development. Just for a brief overview, the topics covered in the WS, EI and DG not only related to questions surrounding the R&D of NL but, aligning with the PARTANE human-rights oriented framework, critically engaged more broadly with current treatment and illness approaches, alternative support strategies, and the epistemic politics in research.

### Participants – who participated?

As described above, the sociodemographic, educational, and experiential backgrounds as well as the years of experience with the topic of R&D of NL were assessed during all AB and WS formats. As shown in Table 1, out of the 34 WS participants, 20 returned this survey data, whereas in the AB meetings, all participants responded to the questions.

As shown in Table 1, the participants belonged to advanced age groups and had many years of expertise in the topic of R&D of NL in both groups. In the WS group, there were more females, whereas male participants dominated the AB. There was overlapping expertise in both groups (lived experiences plus research expertise); the AB meetings hosted more self-identified researchers, whereas more WS participants were identified as users/survivors. Interestingly, one-third of the WS participants succeeded in abruptly discontinuing NL, which is usually not recommended in current guidelines.

### Contents – what was discussed?

The findings of the different participatory formats are presented in detail in a second publication currently under development. In short, the various exchanges during the conceptual phase laid the foundations for the development of the subsequent review design, influencing decisions on the refinement of the main question and development of the sub-questions, the selection of study types and other materials to be included, the decision on the population (age, reconsidering stability as a criterion), interventions (users’ agency and self-determination, reduction strategies, non-pharmacological support, and duration), and outcomes (reconsidering crisis or clinical assessments as criteria). To exemplify how the topics discussed shaped the design of the future review study, a table in the electronic annex (e5) presents the PICO-decisions (population, interventions, comparators, outcomes to be included) that emerged in the context of the PARTANE study from both literature-findings and the discussions during the various participatory activities.

Furthermore, using the above-described methodology, the code distribution across the various participatory formats was tabulated and can be found in Table 2.

In summary, Table 2 demonstrates a declining number of codes on the research topic (R&D of NL) alongside the participatory formats of WS > AB; thus, in the WS, the participants focused their discussions on their own experiences with processes of R&D of NL but also concentrated on the role of their informal caregivers, professional staff, and other circumstances during these processes. Further, a large proportion of the discussions during the WS focused on a multi-perspective exchange of the various reasons for taking and/or tapering these psychopharmacological substances.

**Table 2:** Results of deductive coding of WS and AB transcripts

	3 x WS	4 x AB
Total	100 % (290)	100 % (191)
**Research Topic**	**53,8 % (156)**	**9,9 (19)**
Desiderates in this field	6,9 %	1,6 %
(Own) Experience with reduction / discontinuation	16, 6%	1%
Role of user and survivor knowledge for tapering	2,1 %	1,6 %
Reasons for tapering	5,9 %	0,5 %
Reasons for taking NL	4,5 %	0%
Role of relatives / professionals / circumstances	7,9 %	3,1 %
Need of tapering strategy/guidelines	5,2 %	1,6 %
Lack of information/misinformation	4,8 %	0,5 %
**Methodology**	**37,2 % (108)**	**70,2 % (134)**
Goals of the research	4,1 %	4,2 %
Terminology / definitions	2,8 %	13,1 %
Selection of the appropriate review design	1,7 %	4,7 %
Peer reviewed literature to be included	5,2 %	11%
Approach to grey literature	2,8 %	7,3 %
Population	4,8 %	5,2 %
Intervention	3,4 %	8,4 %
Synthesis of material	1%	1,6 %
Outcome	3,8 %	8,9 %
Limitations and critique of studies	7,6 %	5,8 %
**Collaboration**	**6,6 % (19)**	**19,4 % (37)**
How to do a participatory review	1,7 %	6,8 %
Methods of user involvement / survivors’ knowledge	1,4 %	9,4 %
Epistemological politics / assimilation / cooptation	3,4 %	3,1 %
**Outputs / Dissemination**	**2,4 % (7)**	**0,5 % (1)**

Absolute numbers = absolute frequency of a codes across all WS/ AP meetings. Percentages = relative frequency of a code, adding up to their total percentage of a given sub-section (i.e. research topic, methodology, collaboration)

In contrast, methodological considerations for the future review design were exchanged during the AB meetings, with a focus on discussions of terminologies and definitions, followed by debates on the peer-reviewed literature and possible P(opulation), I(intervention), and O(utcomes) criteria in the subsequent review study, as well as on how to methodologically achieve in including the various sources of users/survivors’ collective knowledge. However, during the WS meetings, a large proportion of the discussion was devoted to methodological aspects, yet in this case focusing on the limitations and critique of existing studies as well as the goals of the current research, whereas a substantial proportion was also devoted to the peer-reviewed literature to be included, possible PIO criteria, and themes around the inclusion of the users/survivors’ knowledge.

### Perceptions – how was this exchange experienced?

#### Evaluation of the standardized survey items

The results of the descriptive analysis of the standardized survey items are shown in Table 3 (scale 1, not satisfied; 5, very satisfied). The items that refer to can be found in the electronic annex (e2).

**Table 3:** Findings from the standardized evaluation of both AB and WS formats

		**3 ×WS**	**4 ×AB**
**General Conditions**			
	Preparation	3,9	4,5
	Understanding of Purpose	3,4	4,2
	Communication	3,9	4,4
	Duration	3,7	4,2
**Inclusion**			
	Inclusion Participants	4,3	4,4
	Inclusion Team	4,4	4,5
	Diversity	3,7	4,5
	Vulnerable Voices	3,6	X
**Own Contribution**			
	Freedom of Expression	4,2	4,3
	Contribution	3,9	3,9
	Satisfaction	4	4,3

Due to a shortage of DG meetings, the perceptions of the participants were not evaluated

Overall, positive evaluations were rated by both the AS and WS participants. The processes of the AD meetings (preparation of formats, the participants’ understanding of their purpose, the clarity and transparency of the research team’s communication, and whether there was enough time to discuss all important questions) were rated more positively compared to those of the WS meetings, and the absolute number of participants was too low to substantiate these differences by a formal analysis. In both cases, the participants apparently achieved to speak freely and felt heard by both the other participants and the research team, contributing to a rather high rate of overall satisfaction.

#### Evaluation of the qualitative material

As stated above, the focus group evaluation involved participants of the AB meetings, yet three of them also participated in one of the three WS. Thus, the following citations mainly refer to AB meetings, further down also relating to WS meetings, which will then be highlighted (see E4 in the electronic annex for the coding system).

Overall, the atmosphere during the AB meetings was perceived to have been good, so the participants enjoyed their participation. Part of this satisfaction resulted from the “idealism” of the research team, making the participants return to the meetings, despite some challenges further detailed below:

*The reason why I kept on coming is because it’s a very nice team. I can see that you are very idealistic. You very much want to do something useful. (F(ocus)G(roup))*

*I liked the appreciative attitude towards personal and difficult experiences within the [workshop] group and also on the part of the research team (S(urvey))*

Further, in both the open questions of the survey and the focus group evaluation, the “diversity of views and standpoints” have been acknowledged. Thereby, the mixed composition of involving both researchers with and without own and collective experiences with R&D of NL in the context of a single AB meeting was described to be “unusual”; apparently they usually are involved in separate groups. Other participants perceived this composition as “refreshing” and to opening the possibility to contribute with their various expertise:

*… I was able to contribute with my both hats on, both as somebody who has used and educed antipsychotics and also as somebody who knows about research. (FG)*

Furthermore, as also shown in the analysis of the standardized survey items above (for both the AB and WS), the purpose of the pre-study phase was not clear to all participants. This lack of clarity added to some confusion, resulting from the way, both the AB and WS meetings of the conceptual phase were implemented, perceived to be far less structured, straightforward, or “directive” than other participatory formats that the participants had experienced previously:

*… we were trying to find out what it was we were doing and how we could contribute and what the different voices around the table felt like. (S)*

*… compared to systematic reviews I’ve been part of in the past, meetings about: what are the inclusion criteria, what are the exclusion criteria? Do we use this or that quality assessment tool? […] You know, it’s a series of quite concrete questions where … we did also talk about that a bit, but it was much broader. (FG)*

Thus, whereas this kind of involvement during the AB and WS meetings was perceived by some participants as having been “too nebulous”, “too participatory”, “too determined to include everybody’s voice” for “too long”, others described these processes to be also productively “meandering”:

*… the discussions we had tent to be non-linear, meandering and messy, which I liked very much. I like messy stuff because things are complex. And this occurs when you mix different audiences and you do not have a specific agenda […] sometimes, it is not as linear, and I think that’s very good. (FG)*

*… some other advisory group meetings felt to me like a rubber-stamping process rather than a genuine sort of interest of what I had to say. (FG)*

*… unless confusion leads to complete paralysis, I think confusion and discomfort with confusion leads to people being less closed-off and to be outside their comfort zone … discomfort is a source of knowledge. If you can work with it, it can be productive. (FG)*

Such an open form of engagement led some participants to ask what contribution they were making to the review design or, more fundamentally, to the success of the project:

*For me, it remained unclear what my contribution to the project might be”. (S)*

*What will come out of this … . this remains a question to me … how the team will further process my statements (S)*

In addition, various causes were imagined for such a style of involvement: first, the participant imagined this style to stem from more temporal resources than review projects usually have, and second, from the composition of the participants, that is, the involvement of user/survivor researchers, who, in turn, affirmed that they were more used to this kind of exchange:

*… reviews are generally being done in six months to a year […] so the meetings are a series of questions that the people must feed into […] decisions are made constantly, because you have shorter periods of time. (FG)*

*For me, the value of the participation was that things felt open. Having been to user groups, I’m used to things not being so determined and therefore I perhaps was more comfortable with the openness and the lack of a clear structure. (FG)*

*… it also comes down to the wisdom of people’s lived experience. This doesn’t fit within the normal rules of academic reviews. We potentially are on the edge of academic work. Maybe that’s where this confusion, fuzziness, perhaps more grayness came from. (FG)*

As stated in the previous citation, on a broader level, this open form of involvement was discussed in relation to scientific processes as practiced in other research projects: openness and confusion were described as having been only “externalized” in the context of the PARTANE study, usually being “less visible” in the context of another research:

*Usually, people don’t talk about it. They give the impression that they know what all is about, the direct path to truth, and that there is only one path. Or that there is a unique way to get data or whatever. Maybe this is wrong. Maybe, to be constantly confused and outraged, like emotionally unsettled is something which can be productive and produce more dense results which are closer to reality in the end. (FG)*

This was discussed in relation to the nature of psychiatric discourse and practices:

*The psychiatric field is bewildering, you know, making you want to pound your head against the wall … often piles of nonsense, which are completely unhelpful to patients and often harmful to them. So, I identify with your sense of bewilderment and confusion. That’s probably an appropriate response to what’s going on out there. (FG)*

## Discussion

In summary, the various participatory formats of the PARTANE conceptual study involved both users/survivors and researchers with extensive lived experience and/or academic expertise in the field of R&D of NL. As shown in the results section, they laid the foundations for the development of the subsequent review design, influencing decisions on its research questions, the selection of studies and other materials, the population, interventions, and outcomes. In addition to these methodological decisions, a range of other topics were surfaced or discussed in depth, such as questions surrounding epistemic injustice or how to successfully reduce or discontinue NL, some of which will be presented in our second publication in preparation.

Therefore, methodological concerns in preparing the subsequent review study were discussed more during the AB meetings but were also exchanged during the WS, given that a large proportion of the participating user/survivors were also equipped with scientific expertise. Overall, satisfaction with participation was high, and the participants also valued the possibility of contributing to the qualitative evaluation. The, to most participants, unknown format of a conceptual phase led to some confusion as well as the rather non-directive way of implementing the participatory exchange by the research team, the latter, at the same time, experienced to do justice to the complexity of the topic of concern and allowing for the effective and self-determined participation of a variety of voices and narratives.

### Participation in an early phase of a review study

None of the few existing studies on how to implement the involvement of various stakeholders in the context of a review study [4, 20, 21, 24, 29] focuses on an early, pre-protocol phase. This manuscript is innovative in this context, as funding models of participatory conceptual phases to plan and prepare review studies together with relevant stakeholders seem to be rare, both worldwide and in the context of the German funding system. This stands in contrast to long-standing claims for early funding possibilities to collaboratively plan and co-design participatory clinical and other research [10, 49, 50], as sufficient time is required for the identification and reach of the relevant stakeholders, joint conceptualization of the design, and cooperative definitions of goals, outcomes, and process. As shown in our study, effective participation requires complex and long-lasting exchange and negotiation processes, both of which require time and resources [51].

Against this background, it is no surprise that none of the participatory review studies that we identified have involved stakeholders intensively during an early stage, at least on what could be inferred from the publication. During the planning of our conceptual phase, this meant a lack of illustrative examples, leading to a plethora of questions at the beginning of our project: how to collaboratively co-design a review study together with various stakeholders, part of them without academic expertise, how to identify these stakeholders and how to reach them, what level of knowledge and agency can be expected on their side, what are their own interests in participating in such a project, and which parts of a review design can be realistically discussed with them to which methodological details? As stated in the introduction and methods section, the experiences of some team members with participatory processes in the context of other studies or projects (UKM, JL, SvP) as well as some user/survivor literature [52] provided for some orientation. Further, some inspiration could be drawn from the two research protocols of future participatory review studies [22, 25, 28], leaving space for imagining whether the participatory steps described had worked out as planned.

This lack of (published) knowledge on how to be involved in the early phase of a review study is surprising, as most advice places particular emphasis on either the early or late steps of a review process. This usually called “top-to-tail” participation [24] is based on the insight that decisions taken in the early (and late) phases of a review project have a high influence on the entire project: the methodological framework is formed during this period, the basal research questions are defined, and the outcomes and other procedures are decided upon, choices that cannot easily taken back once a research protocol has been published. In this context, it was rather unexpected to us that none of the identified participatory review studies presented details on the question if stakeholders had already been involved during the development of either the research proposal or protocol, the “top” already starting at these early stages, laying the fundamental grounds for further decisions and proceedings.

### Composition and goals of the participatory formats

As demonstrated by our results, during the PARATNE conceptual study, the different participatory formats fulfilled various goals: the AB sessions focused more on the exchange of methodological decisions and knowledge, with an emphasis on questions of terminology and definitions, followed by decisions on the literature, population, interventions, and outcomes to be selected during the future review study. In contrast, the exchange during the WS was more diversely distributed, also concerning methodological questions, and here more focused on the critique of existing studies and the user/survivor materials to be included, at the same taking-up in more detailed lived experiences of the R&D of NL, for instance, covering discussions on the reasons for either tapering or taking these substances or the interrelations with professionals or significant kin in this context.

Such a distribution of knowledge alongside the different participatory formats seems to be logical given their compilation: as described, the AB participants disposed of more academic expertise in comparison to those of the WS, whereas a significant number of participants in both formats self-identified as both researcher and user/survivor, explaining the blending of topics. Thus, and perhaps not surprisingly, the knowledge generated during participatory engagement depends on which stakeholders or groups of stakeholders have been sampled for this purpose. Reflexive decisions on whom to involve for which purpose and what kind of knowledge to obtain from these decisions should therefore be invested during the planning of participatory review studies. As trivial as this finding may be, such rationales are usually either omitted completely or presented insufficiently in the above-mentioned identified studies, perhaps also due to the lack of precise terminologies or concepts for their definition [53].

In our case, across all participatory formats of the PARATNE conceptual study, an overarching focus was directed at the involvement of the collective user/survivor body of knowledge. The collective nature of this knowledge in the field of the R&D of NL has been discussed widely [54] and will be at the focus of the planned second manuscript from the PARTANE study. This focus is justified by both ethical and methodological reasons: methodologically, this knowledge has been silenced for decades, leading to a comprehensive but so far in rather neglected body of evidence in both academic discourses and clinical practices; thus, it is expected to produce highly innovative insights in relation to the topic of concern. Mixing these unique positions, having grown globally and differentiated over the years, with other perspectives, for example, through the involvement of professional staff or informal caregivers, would have diluted it and was thus rejected in the current project. Following a more democratic-ethical reasoning for this participatory focus [55] the epistemic injustice of this long-term silencing has justified this priority on user/survivor knowledge: as these are those who are most affected by the intake of NL and their (undesirable) effects, so that their knowledge should be at the heart of any discussions and research on this group of substances.

#### The value and shortcomings of “meandering” participation

As described, the participants have experienced the implementation of the AB-formats and, to some extent, also of the WS – as shown above, some quotes from participants that had experienced both formats highlighted that the style of exchange was comparable – as rather non-directive. As briefly mentioned in the method section, this was intentionally or changed during the project, after we observed that the participants of both the AB and WS needed considerably more time for unrestricted exchange. After preparing the first WS in Germany rather meticulously in terms of content and rather tight scheduling, we switched to this more open style of moderation as it became evident soon, as shown in more detail in the second publication that the participants were very committed to engaging with highly differentiated, well-elaborated bodies of knowledge. In this context, too much pre-structuring this exchange from our side was not only unnecessary but would have severely disrupted this dialogue and multi-perspectivist processes of gradually reaching insights.

Thus, in both the AB and WS, it became clear that the participants were experts, while we tried at best to keep common thread through these rounds of complex, diverse exchange, at times bringing back the discussion to methodological questions but otherwise either listening or joining with their own contributions. As mentioned by some participants, this rather “meandering” style of moderating allowed for various opportunities for them to engage and shape the discussions in a more self-determined way. It involved a less straightforward, formalized set of questions or a fixed agenda, as this seems to be the case in other so-called participatory processes – according to our participants – instead allowing the participants to set their own themes in their own logic, contributing knowledge that was important to them alongside their own interests and needs.

Thus, our non-directive proceedings enabled a nuanced, reflexive exchange of diverse narratives, positions, and perspectives of both AB and WS participants, providing new insights both in relation to the research topic and for the planning of the subsequent review project. On the other hand, as shown in the qualitative evaluation – apparently more suited to collect more critical assessments –, it escalated the level of complexity, making it more difficult to follow. In this context, some participants have mentioned that they did know to what they had contributed, making clear that the purpose of our exchange was not clear to anyone. Even though – to our surprise and delight – the PARTANE evaluation did not bring up the critique on power differentials or tokenistic use of the knowledge produced that often pertains participatory processes [56], such feedback must be taken seriously as potential indicator of a perceived lack of control over outcomes on the side of the participants.

At the same time, this confusion and the discomfort emerging from it was linked to the highly complex research topic, the R&D of NL, which is no clear-cut but related to a range of various questions that are interlinked to each other. Further, the felt confusion was described to also have productive sides: in correspondence with contemporary theories on the production of knowledge [48], some participants described our meandering style of engagement to bring forth new forms of knowledge, to carve out insights and positions previously unknown. Leaving rather formalized ways of thinking and interaction, focusing less on concrete results but more on the dialogical process, thus, seems to open the path to new understandings. It may enable collaboration on more equal terms, allowing for joint processes of exploration in which each person can contribute according to their possibilities, knowledge, and willingness.

o our surprise, the assessment did not involve the usual critique of participatory processes that you have kindly mentioned (power dynamics/tokensim, representational limitations). Yet, there were some sentences in the open survey part that made clear that not all participants understood to what they had contributed. This is now mentioned in the discussion section, also relating to questions of power.

## Limitations

As stated in the method section, the evaluation of the participatory activities was only one out of four work packages within a rather moderately financed, one-year project. Thus, its scope and findings must be perceived of a rather exploratory nature, leading to various shortcomings. Due to the above-mentioned (Section “Reaching out and sampling and selection of participants”) methodological and ethical reasons, the PARTANE evaluative sub-study focused only on material from the AB and WS meetings. Thus, the kind of knowledge produced during both EI and DG was not be assessed systematically, leaving a substantial part of the participatory activities of the conceptual phase unexplored. Second, the perceptions of only three WS participants could be qualitatively assessed that, in addition, were most probably intermingled with impressions on the AB meetings, which had taken part in both participatory formats. This limitation was also for methodological reasons, particularly being linked to the question of whether qualitative evaluations of a one-time event are of any analytical value [49, 50]. Third, the scope of the PARTANE evaluation did not allow for a systematic assessment of the involvements’ impact on the future research design, as carried out in comparable evaluative studies [43]. Such an assessment was also hindered by the fact that the current manuscript focuses on the study’s participatory processes, whereas the second publication one under development presents their outcomes, preventing an interrelated discussion of both in the context of current manuscript. Fourth, as stated in the method section, the involvement of more participants with racialized identities was a major shortcoming, which unfortunately is true for many participatory projects in the field of mental health [51]. Further, as discussed in the second manuscript under development in more depth, the individuals involved were rather of old age and high performing in relation to literacy and academic competencies, also restricts the generalizability of results. At the same time, many focus group and survey participants feedbacked a diversity of standpoints and perspectives among their co-participants, indicating that a range of perspectives on the topic of R&D of NL may still have been involved. Fifth, the limited resources of our project did not allow for a formal validation of our survey instrument nor for an assessment of inter-national differences in research results – the latter mainly due to the limited number of participants in each country.

## Conclusions

To summarize, this one-year preparatory phase proved to be highly useful for the preparation of a participatory, systematic, mixed methods review. It was well-suited to solving the many complex design questions that come with the planning of such a project. As shown above, the “double identities” of the participating users/survivors that also identified as researchers or vice versa – a phenomenon that certainly may not occur in all research fields but is related to the long tradition of user/survivor research in the field of mental health [6, 52] – was helpful in this context, most participants in our participatory formats not only contributing with lived experiences but also methodological expertise. Thereby, this preparatory year not only concentrated on the discussion of “dry” or technical, methodological decisions, instead providing for sufficient time to extensively explore the stakeholders’ specific perspectives and positions to be better able to anchor the design of the future review study in them.

Thus, it was not difficult to fill the entire year with preparations for a participatory, mixed methods, systematic review project. This finding could have certainly been due to the complexity of our research topic, the R&D of NL being a highly complex intervention with many components, discussed continuously and highly controversially over decades in both academic and user/survivor discourses and communities. At the same time, we suppose that the evaluation of any intervention of a more complex nature is suitable for such a comprehensive preparatory phase, providing sufficient time to understand the various and potentially divergent perspectives and positions, and to have enough time to implement the complex processes of participatory exchange and negotiation [57].

## Electronic supplementary material

Below is the link to the electronic supplementary material.

Supplementary Material 1

## Data Availability

No datasets were generated or analysed during the current study.

## Declarations

### Concent for publication

A consent for publication was given by all participants.
