# Supplementary material for: Dialogical meanderings - evaluation framework and learnings from stakeholder involvement in the co-design of a participatory systematic review
Source: Res Involv Engagem. 2025 Nov 26;11:138. doi: 10.1186/s40900-025-00809-w (PMC12659385; doi:10.1186/s40900-025-00809-w)
Supplement: Supplementary file 1 — Supplementary Material 1 [file 40900_2025_809_MOESM1_ESM.docx]

**Electronic Annex**

**E1: GRIPP Checklist – Short Form**

| **Section and topic** | **Item** | **Reported on page No** |
| --- | --- | --- |
| 1: Aim | Report the aim of PPI in the study | pp 5 & 6 |
| 2: Methods | Provide a clear description of the methods used for PPI in the study | pp 5 & 6 |
| 3: Study results | Outcomes—Report the results of PPI in the study, including both positive and negative outcomes | This is the primary topic: Whole results and discussion section |
| 4: Discussion and conclusions | Outcomes—Comment on the extent to which PPI influenced the study overall. Describe positive and negative effects | This is the primary topic: Whole results and discussion section |
| 5: Reflections/critical perspective | Comment critically on the study, reflecting on the things that went well and those that did not, so others can learn from this experience | This is the primary topic: Whole results and discussion section |

**E2: Standardized Survey items AB Participants:**

After the first and second rounds of AB meetings, an online link with these questions will be send to all members of the AB (evaluation on a Likert Scale 1-5):

- - I had enough time to prepare for the meeting.
  - I had a clear understanding of the purpose of this meeting.
  - It was clearly established what end-products had to be produced.
  - The communication by the research team was transparent and clear.
  - I had enough information to contribute to the topic being discussed.
  - The support I needed to participate was available.
  - The time during the workshop was sufficient to discuss the relevant topics.
  - My perspectives were taken seriously by the other participants.
  - My perspectives were taken seriously by the research team.
  - The voices/ perspectives involved were sufficiently diverse.
  - I was able to express my views freely.
  - I could add perspectives/ arguments that otherwise would not have been heard.
  - I felt valued as an expert in the field of tapering NL.
  - I gained new perspectives, knowledge, or skills myself through participating in this meeting.
  - Overall, I feel confident that my input will be used by the research team.
  - Overall, I feel that I made a difference to the research process.
  - Overall, I am satisfied with the process of involvement during this meeting.

**E3: Standardized Survey Items WS Participants**

After each workshop, the participants fill out a short, standardized questionnaire (paper pencil and/ or online?) with these questions (evaluation on a Likert Scale 1-5):

- - I had enough time to prepare for the workshop.
  - I had a clear understanding of the purpose of this meeting.
  - It was clearly established what end-products had to be produced.
  - The communication by the research team was transparent and clear.
  - I had enough information to contribute to the topic being discussed.
  - The support I needed to participate was available.
  - The time during the workshop was sufficient to discuss the relevant topics.
  - My perspectives were taken seriously by the other participants.
  - My perspectives were taken seriously by the research team.
  - The voices/ perspectives involved were sufficiently diverse.
  - Voices from vulnerable, usually excluded, and marginalized groups were sufficiently included.
  - I was able to express my views freely.
  - I could add perspectives/ arguments that otherwise would not have been heard.
  - I felt valued as an expert in the field of tapering NL.
  - I gained new perspectives, knowledge, or skills myself through participating in this meeting.
  - Overall, I feel confident that my input will be used by the research team.
  - Overall, I feel that I made a difference to the research process.
  - Overall, I am satisfied with the process of involvement during this meeting.
  - Overall, the participation in this project has fostered my confidence to contribute to science.

**E4: Coding system**

Composition of participants

Effects of mixed composition/ dual roles

Diversity of participants

Own engagement

Motivations to engage

Challenges to engagement/ perceived barriers

Involvement processes

Abilities to contribute

Feelings of being heard

Overall atmosphere

Overall satisfaction

Performance of the research team

Understandings of involvement

Open/ non-linear involvement

Causes and effects

Directive involvement

Causes and effects

Contextualization of understandings

Reflections on science

Reflections on psychiatric discourses

Benefits of the project

Gain of new perspectives/ knowledge

**E5: PICO-Decisions for the Future Review Study**

| **Literature findings** | **WS, DG, EI and AB findings** | **Plannings for the review** |
| --- | --- | --- |
| **Population:** | | |
| Both quantitative and qualitative studies in the field focus on the population of schizophrenia (F20) or schizoaffective disorders (F25). A few studies also extend to delusional or brief psychotic disorders, excluding drug-induced psychotic disorders. | The restriction of the planned review to one diagnostic group was incongruous to most of the WS participants, especially as NL are prescribed across different groups and often to non-psychotic users (high rate of off-label administration). Given the need to achieve solid evidence, psychosis-related disorders were agreed upon. It was highlighted that polypharmacy is common and part of everyday reality. | Studies will be included on users with F2X.X spectrum disorders and of adult age able to consent. Studies with prescribed medication alongside NL will not be excluded. Observational and qualitative studies/ grey literature will be included if findings can be extracted specifically for users with F2X.X diagnoses, experimental studies will be included if a minimum of 70% of users recruited have a F2X.X diagnosis. |
| **Interventions:** | | |
| The R&D of NL is a complex intervention, entailing various treatment procedures with varying rationales. To present only some examples, the REDUCE study (63) employs a gradual 25% dose-reduction strategy every three months, in some cases until complete D, plus an evidence-based intensive recovery treatment approach. The HAMLETT trial employs existing D schedules that are based on the rationale of a gradual lowering of serum levels of NL, to be tailored in collaboration with the users and their kin (64). The GARMED study employs a need-adapted dose-reduction algorithm as well as a shared-decision-making process to monitor and negotiate the steps of R. In the RADAR trial (65), a standardized protocol to a need-adapted, gradual R is employed that had been developed in a participatory way. In most of these more recent studies, a hyperbolic dose reduction strategy is used to minimize the negative impact of the dopamine-hypersensitivity and related withdrawal symptoms. | Some WS, EI, and DG participants advocated abrupt D if other strategies to compensate are available. Most were in favor of gradual D. Some felt that long-term interventions are necessary for the R&D to be successful, partly over years. Most underscored the importance of supportive networks and professional assistance to deal with symptoms or conflicts behind them. A wide range of support was discussed, and its individual fit emphasized. Self-determination on whether and how to reduce or discontinue NL was advised. | The review will include studies, whose interventions consist of a planful dose-reduction strategy (e.g. the gradual R over weeks or months) up to complete D both with and without additional measures of support (e.g. recovery groups, peer-to-peer consultations etc.). “Planful” means a systematic, purposeful reduction strategy that is either proposed to or negotiated together with users of psychiatric services. |
| **Comparators:** | | |
| The diversity of existing interventions is also true for their comparators: the comparator group may receive the same complementary support as the intervention group (REDUCE), or TAU according to national guidelines (HAMLETT). At times a smaller amount of dose reduction is allowed (HAMLETT), at times the R&D is compared to maintenance therapy (GARMED, 66). | The question of suitable comparators was secondary to the WS participants, a few arguing that a comparison of R&D to maintenance was pointless. | As questions on suitable comparators were subordinated in our WS and only apply to controlled studies, no exclusion criteria will be employed. |
| **Outcomes:** | | |
| Various outcomes, proxies, or theoretical concepts are used in literature to define/ measure the success of the R&D of NL. Quantitative studies and systematic reviews usually focus on relapse rates, definitions of which vary. Some recent studies prioritized quality of life as primary outcome, among them only one recent RCT. There are a variety of secondary outcomes, among those symptom severity or social functioning. Qualitative studies explore experiences to define or assess the success of the R&D processes, less focusing on ‘end-results’ or pre-defined outcome measures. | A huge part of the dialogue concentrated on the question of what success means in the context of the R&D of NL and how it may be assessed. It was argued that relapses must be seen as an integral part of R&D process and not as a (negative) outcome. Alternatives were discussed into depth, such as increased recovery or well-being, improved abilities to think, feel or work, or more self-determined administration of NL, all of them being discussed as being both outcomes and possible mediators of this complex intervention. | In the context of the planned review, “success” will be used as an explanandum instead of an explanans. Collecting and analyzing the various conceptualizations and definitions existing will be part of both the quantitative and qualitative data extraction. The quantitative component will consider outcomes related to the course of illness (e.g., relapse, readmission), to adverse events (e.g., withdrawal syndromes), and patient-reported outcomes (social functioning, or quality of life). Studies that use intervals between intervention and outcome measurement will be included although the primary time point of interest is 1-2 years for most outcomes. The qualitative component will consider studies that investigate experience of the R&D of NL, focusing on user perspectives, preferences, satisfaction, expectations, and attitudes. |
